# Supplementary material for: Transient Vasodilation in Mouse 4T1 Tumors after Intragastric and Intravenous Administration of Gold Nanoparticles
Source: Int J Mol Sci. 2021 Feb 26;22(5):2361. doi: 10.3390/ijms22052361 (PMC7956783; doi:10.3390/ijms22052361)
Supplement: Supplementary file 1 [file ijms-22-02361-s001.pdf]

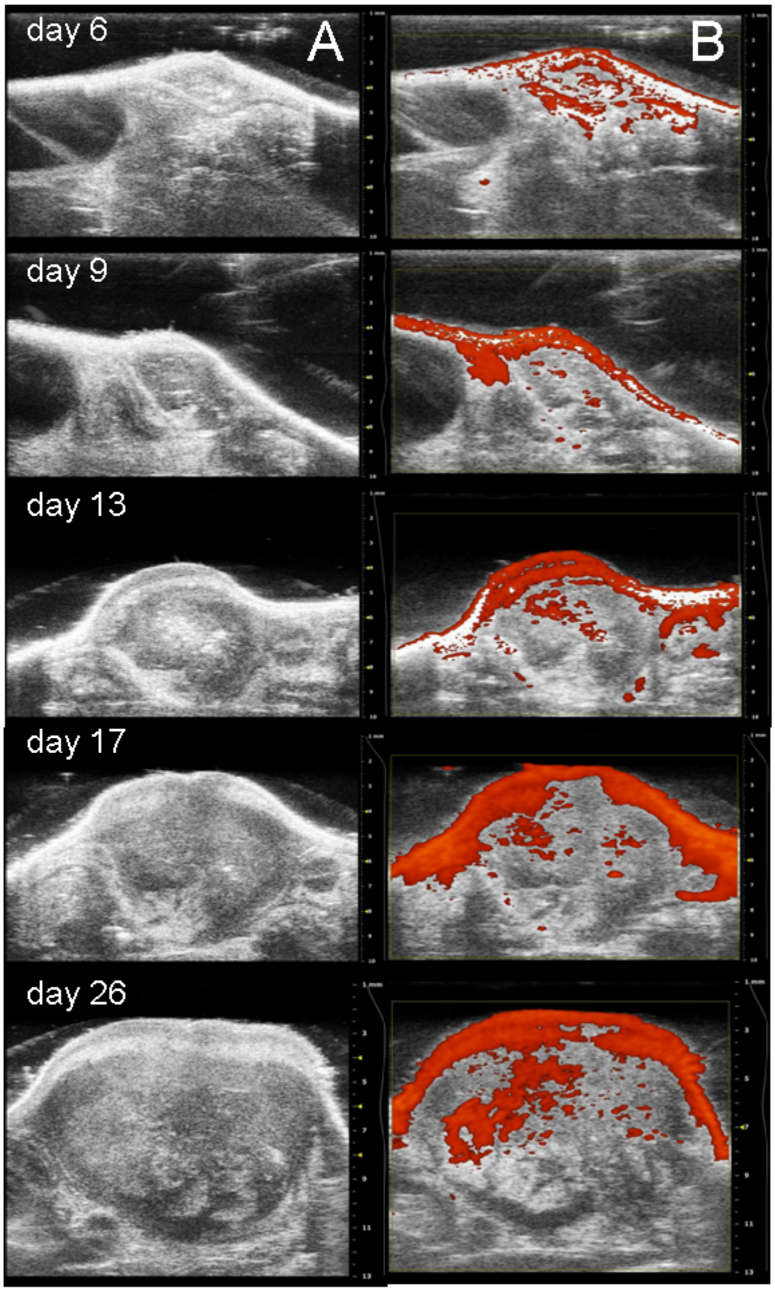

Figure S1. Quantification of the tumor volume and the volume of tumor's blood vessels. A sequential USG pictures were taken every 200–400 micrometers at the tumor site combined with the measurements of blood flow by Doppler technique. A 3D tumor model was built based on these pictures, and the tumor volume and the volume of area containing blood vessels were determined. (A) Exemplary USG pictures of T41 tumor (mouse no. 98) at day 6, 9, 13, 17 and 26. (B) Areas characterized by blood flow, as measured by Doppler technique, are shown in red.
